# Exercise therapy as a digital therapeutic for chronic disease management: consideration for clinical product development

**DOI:** 10.3389/fdgth.2023.1250979

**Published:** 2023-12-20

**Authors:** Andrew Grannell, Hallur Hallson, Birkir Gunlaugsson, Hedinn Jonsson

**Affiliations:** Sidekick Health, Research & Development Unit, Kópavogur, Iceland

**Keywords:** digital therapeutic, exercise therapy, chronic care, wearable sensor, medical device

## Abstract

Digital exercise therapies (DET) have the potential to bridge existing care gaps for people living with chronic conditions. Acting as either a standalone, embedded within multi-modal lifestyle therapy, or adjunct to pharmacotherapy or surgery, evidence-based DETs can favorably impact the health of a rapidly growing population. Given the nascent nature of digital therapeutics, the regulatory landscape has yet to mature. As such, in the absence of clear guidelines clinical digital product developers are responsible for ensuring the DET adheres to fundamental principles such as patient risk management and clinical effectiveness. The purpose of this narrative review paper is to discuss key considerations for clinical digital product developers who are striving to build novel digital therapeutic (DTx) solutions and thus contribute towards standardization of product development. We herein draw upon DET as an example, highlighting the need for adherence to existing clinical guidelines, human-centered design and an intervention approach that leverages the Chronic Care Model. Specific topics and recommendations related to the development of innovative and scalable products are discussed which ultimately allow for differentiation from a basic wellness tool and integration to clinical workflows. By embodying a code of ethics, clinical digital product developers can adequately address patients' needs and optimize their own future digital health technology assessments including appropriate evidence of safety and efficacy.

## Introduction

The digitalisation of healthcare systems and clinical care has progressed at an underwhelming rate (1). The COVID-19 pandemic reminded us once again of a universal truth: “Necessity is the mother of invention”. Under the cloud of lockdowns, social distancing and a strained system caring for the most vulnerable, the need for remote solutions was catalysed (2). One such solution is digital therapeutics (DTx) which is defined as an adjunct or standalone “evidence-based therapeutic intervention that is driven by high-quality software programs to treat, manage, or prevent a disease or disorder” (3). DTx solutions are positioned to favourably impact healthcare by bridging treatment gaps, improving access to care and enhancing clinical outcomes (4). When successfully embedded into healthcare provider workflows, the clinical outcomes can be realised through remote patient monitoring, patient education, prescription of multi-modal care and data-driven adjustment of the therapeutic regime.

The DTx is a clinical product that must go through phases of ideation, needs analysis, development (defining, prototyping and designing), testing, marketing strategy, commercialisation, iteration, growth, and maturity. However, first and foremost, at its core, the product is a clinical treatment and thus must adhere to fundamental principles of clinical care (5). Indeed this is what distinguishes the DTx as a true treatment and prescription tool as opposed to a wellness product promoting generalised health advice. As such, the product should be built through human-centred design with a key focus on safety, clinical impact, the durability of effect via self-care, usability & accessibility and user engagement in order to demonstrate treatment effect through clinical and real-world evidence (3, 6).

Currently, there is a paucity of clear and consistent regulatory pathways and digital health technology assessment frameworks in place across countries (7). In the United States, the FDA has positioned DTx solutions under the same category as hardware-based medical devices, framing them as software as a medical device, subject to regulatory approval, which may not be fit for purpose (8). This may prove a hindrance as the DTx utilises continually improving cutting-edge technology, and thus innovation and iteration of products are inevitable. The need for a new adaptive regulatory paradigm has been acknowledged by the FDA with the Digital Health Software Precertification Pilot Program highlighting the need for legislative change that will take time to occur (9). In Germany, The Fast-Track Process for Digital Health Applications (DiGA) has been implemented to accelerate into the market “apps on prescription”, which are considered class I or IIa medical devices (10). Here the DTx has the opportunity to enter the market on a provisional listing prior to the demonstration of clinical evidence for a positive healthcare effect, although this must be achieved to retain status within the DiGA directory. The National Institute for Health and Care Excellence has developed an evidence standards framework for digital health technologies which enables both evaluators and decision-makers in the healthcare system to identify technologies that offer benefits to key stakeholders and the healthcare system (11). Within this framework, a DTx (Tier C) is differentiated from a wellness tool (Tier B). Here a Tier B digital health technology (DHT) simply promotes good health via non-personalised information and resources to service users, encouraging behaviors that promote good health and address issues such as smoking, nutrition and exercise and providing information about specific conditions. In contrast, a Tier C DHT, which is broken into specific sub-types, can diagnose a condition or inform and drive clinical management and as such needs to be supported by evidence demonstrating accuracy and reliability. A Tier C DHT may also treat and manage a condition where information provided by the DHT is used to take an immediate or near-term action to treat, prevent or mitigate by means of providing therapy to a human body.

There exist an overwhelming amount of wellness and fitness-themed apps available to users, many of which present with privacy concerns and an absence of clinical evidence (12). Digital exercise therapy (DET) distinguishes itself from these offerings, being supported with clinical evidence and the software having been designed to serve as a standalone or adjunct disease treatment or management tool. As highlighted by the DTx Alliance, there is a need to adhere to a code of ethics when developing these products (13). The first principle of medicine is to do no harm, and as such, tenets of clinical governance such as risk management and clinical effectiveness should be at the forefront when building any DTx. In this narrative review, we aim to highlight key considerations when building a generalisable and scalable DET as a DTx that can be integrated into existing clinical workflows and thus clearly differentiate the product from a basic wellness tool. As DTx product development is still in its infancy, key considerations must be informed by clinical standards of care. To identify standards of care to use as a framework for this review, clinical guidelines related to chronic disease management and exercise therapy were examined. The search strategy included the terms: digital therapeutics, chronic disease management, exercise therapy, physical activity, aerobic exercise and, resistance exercise. The search was completed in Medline, SPORTDiscus, CENTRAL and EMBASE. We identified the American College of Sports Medicine Guidelines for Testing and Prescription (14), the DTx Alliance fit-for-purpose evidentiary standard (3) and the Chronic Care Model (15) which serve as frameworks in this narrative review to highlight key considerations for DET product development.

## Digital exercise therapy: mechanistic considerations

Exercise therapy is a well-established component of multi-modal care in patients living with cardiovascular and pulmonary conditions (16, 17). However, despite clinical evidence highlighting the effectiveness of exercise therapy in most chronic conditions, it is not regularly prescribed (18). This reality exists in spite of the American College of Sports Medicine and the World Health Organisation promoting exercise therapy as not only a preventative tool but also a treatment and disease management tool (14, 19, 20) Typically, exercise therapy is delivered in person by a clinician such as an exercise physiologist, and therefore DET is a treatment tool delivered through software that must adhere to the fundamental principles of traditional care. Exercise therapy traditionally consists of general physical activity, which refers to all waking movement, cardiovascular or aerobic exercise referring to pre-planned continuous movement targeting a specific time and intensity, and finally, muscle-strengthening exercise referring to pre-planned movements which load muscle, tendon and bone (14).

In developing a DET as a clinical product, a hypothesis must be tested. Before understanding what research question to ask, one must develop an understanding of what the therapy achieves mechanistically. In doing so, the DET as a clinical product may effectively scale across multiple chronic diseases. For some chronic diseases, exercise therapy has been shown to directly target the underlying pathophysiology: atherosclerosis (21), hypertension (22), osteoporosis (23) and type 2 diabetes (24). There is no evidence from human randomised controlled trials for this effect in some conditions such as cancer (25) and parkinsons (26). However, in these conditions and beyond, it has been well established that although mechanistically, there is uncertainty regarding the role of exercise therapy in tackling the underlying pathology, there is a clear therapeutic benefit for the patient. This is due to the commonality of complications associated with most, if not all, chronic conditions. Frailty of varying degrees is an inevitable consequence of the ageing process (27). In the context of chronic conditions, the entrainment between biological age and chronological age is lost, with the latter accelerated (28). The result is the vulnerability of the biological system to stressors which increases morbidity and mortality (29). Therefore the primary outcomes in the context of disease agnostic exercise therapy should be to reduce the risk of developing, reversing, or halting frailty by improving or maintaining physical function along with psychological wellbeing. The following outcomes are associated with disease severity, can be measured through easy-to-administer tests face to face and are sensitive to an exercise therapy intervention with favourable changes documented:

- Cardiorespiratory fitness (30, 31)
- Muscle strength (32)
- Muscle function (33)
- Neuromotor control (22)
- Health-related quality of life (34)

**Figure 1:**
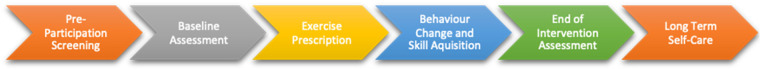
Digital exercise therapy intervention cycle. This model has be utilised to bring about health optimization for people living with a chronic condition. The outcome should be self-care, however the treatment cycle may need to be completed more than once before the patient develops the skills needed to manage independently long term. DET developers must strive to build a DTx solution through human centred design with both patients and clinicians in mind.

Whilst a DET can be framed as a precision tool, directly targeting the underlying pathology in some cases, a scalable product for patients living with ≥1 chronic condition should be built with disease complications in mind. Moreover, the DET is unlikely to be experienced in isolation as it will likely complement pharmacotherapy, surgery and other traditional aspects of care such as nutrition therapy and psychosocial support. Ultimately, by first establishing the mechanistic approach, the right research questions can be asked, and a clinical product that meets the needs of people living with chronic conditions can be developed. This will ensure the initiation of product development takes place with patient needs, risk management and clinical impact in mind. Whilst it is beyond the scope of this paper to detail how to achieve optimal integration in the marketplace, it is essential that product development, early on, considers key stakeholders: both patients and care providers, through human-centered design. Only by keeping these stakeholders in the forefront can a product be developed that has both dual clinical impact and appropriate uptake in a frictionless manner within the healthcare ecosystem it aims to target. The first goal of DET should be to try to achieve the same clinical outcomes as those seen in traditional approaches, thereafter the goal should be to go beyond and ensure the durability of effect. Thus DET product developers must understand the components and ingredients needed to develop a minimum viable clinical product. We will now provide an overview of these components and ingredients which facilitate both risk management and clinical effectiveness. When framed as a personalised intervention (Figure 1), DET has the potential to drive significant clinical impact.

## Pre-participation screening

In a clinical setting, the first interaction with a patient enables appropriate risk management, needs analysis and baseline assessment. This can be achieved through screening questions, motivational interviewing and standardised assessments that enable (14):

1. Identification of individuals with absolute contraindications
2. Implementation of risk stratification based on medical history
3. Detection of symptoms and or risk factors that may activate referral to a medical doctor for evaluation
4. Identification of mobility issues, musculoskeletal injury or disease that may affect exercise testing and programming
5. Understanding the motivations and perceived needs of the patient
6. Understand the clinical needs of the patient

With DET, in the context of implementing an intervention in the absence of a skilled clinician, safeguarding the patient is the priority. As such pre-treatment screening should be risk-averse and begin early on during the onboarding of the user through patient-reported outcomes, questionnaires and decision trees. In a clinical setting, there is an opportunity to engage with the user during a consultation that may last between 30 and 60 min. From a temporal standpoint, this is not realistic for an in-app experience, and thus consideration should be given to the enhancement of engagement. The utilisation of assets to aid pre-treatment screening may depend on the chronic condition(s) being treated. We here present a brief overview of key elements for consideration related to the pre-participation phase of care when building DET products. Importantly, elements of pre-treatment screening may contribute to baseline assessment measures and thus can be utilised as means of examining treatment effects. Deciding on what aspects to utilise should be made through a human-centred design approach.

### Absolute contraindications

In a clinical and, therefore, digital setting, it is important to consider absolute contraindications to exercise (35). Whilst relative contraindications can be examined by the general practitioner where a risk:benefit decision can be made with an emphasis on caution, patients presenting with any of the following are considered unfit to engage in any structured exercise program until their presentation has been brought under control:

- Unstable angina
- Systolic blood pressure ≥ 180 and/or diastolic ≥ 100 mmHg
- BP drop > 20 mmHg demonstrated during exercise tolerance testing
- Resting tachycardia > 100 bpm
- Uncontrolled atrial or ventricular arrhythmias
- Unstable or acute heart failure
- Unstable diabetes
- Febrile illness

In a clinical setting, patients are typically screened prior to commencing any physical activity or exercise program through a physical activity readiness questionnaire (PAR-Q) or electronic Physical Activity Readiness Medical Examination (36). The value of this PRO when working with vulnerable individuals is that it is helpful to highlight potential contraindications to exercise and direct the patient to their healthcare provider where necessary. The PAR-Q has been adapted to increase the likelihood of the individual engaging in exercise as the health benefits of participation far outweigh the risks in the vast majority of asymptomatic and symptomatic individuals (36). Where a DET product aims to achieve regulatory approval as a DTx, there is a need to demonstrate safety. As such careful consideration should be given to how the patient is safeguarded during this initial process. Where the DET is embedded into an existing healthcare ecosystem and is thus prescribed by a healthcare provider, the risk management element of pre-participating screening can happen pre-app prescription. However, where the DET is “over the counter”, there is a need for those building DET products to appropriately consult with HCPs to arrive at an optimal process that ensures adequate safety without burdening care practices.

### Physical activity and exercise status

The international physical activity questionnaire (IPAQ) is a widely used tool to examine activity status. The purpose of the IPAQ is to quantify self-reported activity levels. The short form version records the activity of four intensity levels. Importantly a systematic review examining the validity of the IPAQ-SF found that the correlation with objective measures was below an acceptable standard with an overestimation of 84% (37). The authors concluded that the evidence to support the use of the IPAQ-SF as an indicator of relative or absolute physical activity is weak. It should be noted that within this review article, the authors found that both vigorous and walking activity showed acceptable correlations in some studies against fitness and accelerometer data respectively. Whilst IPAQ is often in research settings, in clinical practice, standardised questions from the clinician enable needs analysis. In addition, the IPAQ is time consuming and, in the context of an in-app experience, it may negatively impact engagement. It is, therefore, important for DET products to focus on the needs of the patient and how simple questions can aid in care management. Integration of wearable data or self-reporting of the patient regarding current physical activity and exercise status can ensure the right treatment for the patient. For example, a patient with cancer cleared to exercise, reporting adequate minutes of cardiovascular exercise with an absence of muscle-strengthening exercise, will logically benefit from an adapted care plan relative to a sedentary patient.

### Identifying relevant conditions and lifestyle factors

In clinical settings, once cleared to proceed, examining health status enables the needs of the patient to be identified, optimization of the therapeutic intervention and subsequent quantification of treatment effect. An examination can include disease status, injury history and status, current sleep quality and quantity, and dietary quality such as protein intake and distribution. Here wearable sensors and smartphone feature development leveraging off-the-shelf technology with evidence to show accuracy and reliability can enhance this process. For example, photoplethysmography can potentially allow for examination of atrial fibrillation (38) whilst accelerometry can potentially be used to monitor tremor status in patients with Parkinsons (39). In a DET context, the emphasis should always be placed on relevance, as excessive and unnecessary data collection is likely to drive disengagement. Therefore, it always depends on the treatment context, which will determine the prioritisation of data collection. Incorporation of human-centred design principles early on in the product development process can ensure relevant and frugal use of digital space during onboarding, screening and assessment. Of relevance, however, are fall risk, pain and fatigue which are common in people living with chronic conditions and come with a substantial economic toll (40–42). Therefore, consideration should be given to examining the effect of the DET on these outcomes for these populations where applicable.

One in four older adults experience a fall each year which can have devastating and long-term consequences, including reduced mobility, loss of independence, and premature death (43). In the US, annually, it is estimated that 36 million falls occur, which leads to 32,000 deaths. As a result, $50 billion is spent on medical costs related to nonfatal fall injuries, and $754 million is spent related to fatal falls (40). As chronic disease prevalence is highest in older cohorts, many patients will benefit from an intervention to reduce fall risk (44). It is, therefore, of clinical interest to screen for risk of falling. This will enable optimal risk management and personalisation and offer an additional metric with which to measure treatment effectiveness.

A meta-analysis of randomised controlled trials of fall interventions concluded that assessing and addressing fall risk factors, in addition to identifying and treating symptoms of chronic conditions, can reduce falls (45, 46). In clinical settings, adults receiving preventative care to reduce the risk of falling do not need to undergo a comprehensive assessment (47). In the context of secondary prevention, a comprehensive assessment is considered necessary prior to undertaking treatment through a validated intervention (48). The Falls Risk Questionnaire (FRQ) is a 12-item questionnaire used for screening older adults who are at risk of falling. The FRQ has been validated against the gold standard clinical assessment of fall risk using the American/British Geriatrics Society guidelines to assess independent predictors of falls and is reliable (49, 50). In addition, the FRQ is positively correlated with the timed up-and-go test, the berg balance scale, and the 5 times Sit-to-Stand test (50). It is logical when building a DET for people living with chronic conditions to consider examining fall risk, quantifying balance ability (which shall be discussed below) and subsequently implementing a fall prevention treatment plan.

Pain is a complex biological phenomenon with diverse etiologies (51). In 2010 it was estimated that 100 million Americans were living with chronic pain, with an estimated cost of up to $635 billion per year (40). Pain is a commonly reported experience of older adults and those living with a chronic condition (52). There exist multiple patient-reported outcome measures related to pain. However, many are joint site-specific or relate to an occupational setting (53). In the context of a DET, there is a need to utilise a PRO that is generalisable to a wider population. Again the value relates to the optimisation of care and quantification of the treatment effect. The brief pain inventory was originally developed as an instrument to examine the experience of pain in patients with cancer regarding the severity of pain and impact on daily functioning (54). It has been applied to several populations and is now used in patients with pain from chronic diseases or conditions such as osteoarthritis and low back pain or with pain from acute conditions such as postoperative pain.

Fatigue is an experience of physical and/or mental weariness with multifactorial etiology (55, 56). The prevalence of fatigue and, moreover, chronic fatigue is likely underestimated due to inconsistent definitions, lack of biomarkers and unwillingness of medical professionals to diagnose it (41). Multiple chronic conditions are associated with perceptions of fatigue (57). Given that fatigue is associated with excess mortality in the general population there is a need to consider antifatigue treatment solutions (58). Identification of fatigue experienced by patients during screening is thus relevant and can lead to the enhancement of intervention strategies. The Functional Assessment of Chronic Illness Therapy—Fatigue (FACIT) was originally developed to assess fatigue in cancer patients with anaemia (59). The full 40 item version covers the broad quality of life: (a) physical wellbeing (b) family/social wellbeing (c) emotional wellbeing (d) functional well being and (e) fatigue. This has been validated beyond cancer to many additional chronic diseases and the general population. Given the deleterious effects of fatigue on health and quality of life, it is important for DET product developers to screen for this at baseline and then quantify it in those presenting with fatigue. This can allow for tailored solutions and appropriate signposting. In addition, it can be used to assess the effectiveness of the intervention. Overall fall risk, pain and fatigue should be at the forefront of the minds of clinicians and product developments when building DETs through human-centered design.

### Shaping engagement and retention

In a traditional clinical setting, the first contact time with a patient is an opportunity to identify predictors of adherence by examining motivation, behavioural history, environment and perceived barriers. A recent umbrella review of key factors associated with adherence to physical exercise in patients with chronic diseases and older adults revealed the following as important determinants (60): Multidisciplinary support, supervision and social support, exploration of characteristics, barriers, and facilitators, treatment clarity and education, enjoyment, integration to daily living, communication and feedback, access to progress information and monitoring, consideration for self-efficacy and competence, a sense of playing an active role and goal setting. There is a wider need to consider all of these factors within the DET product throughout the journey of the user, particularly with regards to behaviour frameworks, behavioural change techniques, coaching interaction, educational assets, exercise prescription and perhaps most importantly, creating an overall sense of meaning. From the perspective of baseline PROs there is a pressing need, based on the literature, to examine self-efficacy to exercise along with the identification of present barriers.

In a clinical setting, there is an opportunity for open questions and rich conversation, which cannot be replicated to the same extent in a digital offering without an embedded clinician interaction feature. Whilst the emphasis on adherence should be present throughout the entire digital intervention, consideration should be given to what should be prioritised early on in the experience of the patient. The Self-Efficacy for Exercise Scale (SEES) was developed in light of the findings that self-efficacy is theorised to influence the types of activities an individual chooses, the effort expended on the activity, and the persistence of one's behaviour when faced with challenges (61, 62). The SEES includes 9–11 items depending on the version employed, which gauges the beliefs of the individuals regarding their ability to engage in exercise for 20 min (some scales 40 min) 3 days per week. The scale has been shown to be valid and reliable in a number of different populations and predicts exercise behaviours over time (62, 63). An adapted home exercise version has been developed with demonstrated internal consistency and convergent validity with the SEES (64). The authors concluded it is a clinically useful tool to evaluate a patient's self-efficacy in home-based musculoskeletal exercise programs. Thus in the absence of an opportunity to examine perceptions and beliefs in detail through conversation, tools such as the SEES should be considered.

## Assessment

The value of a thorough pre-treatment screening process centres around risk management. For example, an individual with uncontrolled hypertension should not partake in a cardiorespiratory fitness test. The purposes of assessing physical performance includes optimisation of the therapeutic care plan and subsequent quantification of treatment effect. In a DET setting, the patient is likely to be remote from the care team. As such significant thought must be given to what assessments are safe and enable quantification of treatment effect. This is of particular importance from a regulatory standpoint, as the treatment must be safe and allow for the capture of real-world data. In the clinical trial phase of the DET product development, traditional clinician supervised tests are likely to be employed. We herein provide examples of basic tests which can be utilised in the real world through off-the-shelf technology found within current-generation smartphones and wearables.

### Cardiorespiratory fitness tests

Cardiorespiratory fitness (CRF) measures the capacity of the circulatory and respiratory systems to supply oxygen to skeletal muscle mitochondria for energy production needed during physical activity (65). Across the lifespan, CRF is an important indicator of health status and function, with longitudinal data showing CRF predicts all-cause mortality (31). Specifically, every 3.5 ml kg min increase in VO_2max_ is associated with an 11% reduction in mortality (31). Age-related declines in CRF have been noted after the age of 45 and are associated with activity status (66). Accelerated changes that occur later in life or in those with a chronic disease increase the risk of advanced frailty status (67).

The gold standard approach to measuring CRF involves the estimation of maximum aerobic capacity (VO_2Max_) through indirect calorimetry. VO_2Max_ measures maximal oxygen consumption during an incremental exercise test (65). A number of protocols exist, but all will see the utilisation of a metabolic cart which enables the quantification of inspired and expired gas. Submaximal tests such as the Astrand Rhyming test and the Ekblom-Bak test circumvent issues with expensive technology as they require minimal equipment. However, currently, these tests are not suitable for a DET employed in the real world.

Wearables offer a potential solution regarding the estimation of VO_2Max_. The Apple Watch along with Fitbit and Garmin devices, all estimate VO_2Max_ from submaximal physical activity heart rate metrics with varying degrees of validity and reliability (68–71). For DET developers, it should be acknowledged that most individuals living with a chronic condition are older adults and thus, a substantial proportion of users will fall into this age category. A recent study of >1,100 Swiss adults >65 years of age found very low levels of smartwatch (3.3%) use (72).

There is a need, therefore, to consider safe, self-administered tests that can be utilised. The 6 min walk test (6MWT) has been used to examine functional status, submaximal exercise capacity and exercise tolerance of individuals with pulmonary disease (73, 74), peripheral artery disease (75), heart failure (76, 77), and healthy, older and elderly individuals (78–80). A recent review suggests the 6MWT may not be accurate in predicting VO_2Peak_ in CHF patients (81), whilst in healthy adults, the 6MWT predicted VO_2Max_ to be within 1 MET (82). In healthy young, to middle age adults, the 6MWT was shown to be a predictor of functional (distance) and objective (VO_2Max_) fitness (83). The minimal clinical important difference for the 6 min walking test across various chronic conditions was shown to be 7% (84).

Whilst originally developed and employed in a clinical setting, technology has provided an opportunity to administer the test remotely. Several remote approaches have been developed, but few have undergone appropriate testing for validity and reliability (85). A remote indoor and outdoor 6MWT has been tested in patients with pulmonary hypertension (86, 87). The authors found suitable accuracy, reliability, usability and acceptance. Interestingly the authors found low compliance to engage in more than 1 remote 6MWT during a 6-month period in 52% of their cohort. They speculated engagement could be diminished if the data is not used for clinical decision-making.

The 6MWT, whilst a logical choice for DET providers, should be developed in a thoughtful manner and strive to be as inclusive as possible for patients. To optimise safety, the 6MWT should undergo appropriate testing to ensure safety, accuracy, reliability and usability. The seminal guidelines from the American Thoracic Society regarding protocol should be considered as a means of optimizing patient safety (88).

### Physical function and neuromotor control tests

Muscle is a key determinant of metabolic health, CRF, strength, power, reactive ability, gait speed, and neuromotor control (89). Peptides produced by skeletal muscle known as myokines mediate many system-wide effects on health through muscle-organ crosstalk (90). Indeed, meta-analysis shows that upper and lower limb strength predict all-cause mortality, and this may be independent of muscle size (32, 91). Muscle mass decreases after the 3rd decade of life, with accelerated changes observed in older adults and those living with some chronic conditions (92). The loss of muscle mass accompanied by diminished function is known as sarcopenia. This condition is most prevalent in the older population and is intricately linked to frailty status.

There are several aspects of muscle health that can be examined: size, quality, strength and function. Similarly to CRF, in traditional settings, myriad tools can be utilised to examine body composition (DXA, MRI) and performance (dynamometry, kinetics, kinematics etc.). Whilst these can and should be employed in DET clinical studies, in the real world, only that which can be measured safely, accurately and reliably should be considered. Although the sit to stand test is not a measure of muscle strength *per se* but rather a test of functional capacity determined by muscle strength and neuromotor control, it represents an easy-to-administer assessment tool (93). Versions of the test have been shown to predict falls (94) and the test has been shown to be both valid and reliable, correlating with muscle mass of the quadricep (95), muscle force/torque (96, 97), static and dynamic balance (98) and gait speed (99). The test itself can be performed where the individual attempts to complete a number of repetitions as quickly as possible from a chair (5 or 10, for example) or the maximum number that can be performed for a given period of time (30–60 s for example). The 30 s version of the test has been proposed as an alternative to the traditional 5 times test as a means to overcome the floor effect observed in some cohorts (100). The test can also be modified whereby the user can use the armrest of a chair to assist in performing the test with outcomes associated with fall risk. For the 30 s version of the test, the minimum clinically significant difference has been proposed to be 2 repetitions (101).

The sit to stand test represents the logical target as a remote self-administered assessment. The safety and feasibility of a telehealth administered 30 s STS as a measure of function and lower limb strength has been demonstrated in patients with cancer (102). A video-led, self-administered 30-s sit-to-stand test was examined in over 1,800 individuals living with cancer (103). The authors found that the remote self-administered, video-guided tool is feasible for implementation within large, longitudinal studies and provides a score that may be useful for understanding participant muscular strength and mobility. With just household items and a smartphone, the range of physical function tests to examine primarily muscle performance is limited. The sit to stand test is a logical target for DET developers.

The ACSM position neuromotor fitness as a core pillar of health, which can be developed in part through muscle-strengthening along with motor skills training, including balance, coordination, gait, agility, and proprioceptive training (104). These motor skills are rarely trained in isolation. For example, static balance exercises can involve balance, coordination and proprioceptive ability. Walking can involve balance, coordination, gait, agility and proprioceptive ability. Thus balance, neuromotor and functional training are terms used interchangeably in clinical practice. A key element of the neuromotor ability which can be favourably impacted in a measurable by exercise therapy is balance ability. This is a key consideration for people living with a chronic condition and can reduce risk of falls. Indeed factors associated with risk of falling include: Chronic disease (105), age (106), impaired vision (106), hearing loss (106), self-efficacy (107), excess adipose tissue (108), muscle loss (109), reduced muscle strength (109), joint degeneration (109) and certain medications (106). These risk factors interact with the environment to increase risk of falling (110). There is thus a need to examine the risk of falling in DETs, examine balance ability and then develop an intervention around enhancing each motor skill and address additional modifiable risk factors to reduce risk of falling long term.

As described previously, non-invasive fall risk assessments can form part of the pre-treatment screening process. There is then a need to examine an individuals balance ability, an element of neuromotor fitness, prior to implementation of an intervention to reduce the future risk of falls. In a traditional setting, risk is reduced by the presence of a trained professional who can safeguard the patient. In a remote DET setting, the absence of supervision increases risk. Whilst clinical research associated with the DET will allow for traditional tests, real-world evidence calls for novel solutions.

The timed up and go test (TUG) is a dynamic test that examines aspects of neuromotor control. Off-the-shelf smartphones with in-built sensors are capable of capturing rich data needed for tests like TUG (111). TUG completion time has been accurately measured via a smartphone app using integral accelerometers and gyroscopes (112). TUG test completion time utilising this same approach has been shown to be accurate compared to stopwatch measurements and physicians' reports (112, 113). Static tests, such as the single leg balance test, has been well established as a predictor of fall risk (114), fracture risk (115) and rate of decline in patients with dementia (116). An association between the ability to perform a 10s single leg balance test and mortality in 1,702 individuals between 51 and 75 years of age has been described (117). However, concern exists regarding the safety of this test for patients in remote scenarios (118)**.** Here smartphone-based static balance assessments were examined with an emphasis on ease of use, safety, and reliability in individuals >65 years of age (118). There was good agreement with force plate regarding kinematics for bilateral stance, tandem stance and single-leg balance stance. Whilst the single-leg task was considered potentially unsafe by the authors, the tandem stance was deemed more suitable and also had fewer invalid tests.

There is currently a paucity of research examining static balance tasks employed in a remote setting. Currently, there has been no app/remote test validated from the perspective of the duration of the task. Whilst more progress has been made with dynamic tasks, they usually require additional equipment to secure the smartphone to the sternum or limb. DET developers should strive to employ safe, valid and reliable balance tests within their assessment suite prior to implementation of the intervention.

In conclusion, there is a pressing need for DET developers to incorporate safe, relevant, valid and reliable assessments in the treatment plan that enable optimization of exercise prescription and quantificaiton of treatment effect. Cardiodespiratory fitness, physical function and balance ability are logical starting points for individuals living with chronic conditions. In addition, there is a need to incorporate quality of life questionnaires. There are myriad implementable solutions which can further enrich the treatment package and the user experience. Of importance is the impact a quality of life measure can have on examining cost-utility, which should be a consideration for DET developers.

## Exercise prescription and patient management

The primary aim of exercise therapy is to achieve clinical impact by favorably influencing the health of patients living with a chronic condition. Mechanistically, one can design an intervention directly targeting the underlying pathophysiology. However, in most cases, for exercise therapy, the target will be the complications of the disease itself. This is an important distinction which facilitates the appropriate use of outcome measures. As discussed, pre-participation screening followed by a battery of tests ensures risk management, personalisation and the formulation of an intervention leveraged to maximise clinical impact.

The traditional approach is based on the dose response relationship between physical activity, exercise and health (31, 119, 120). Exercise therapy guidelines for various chronic conditions have been developed, which propose broad targets for steps per day, minutes of cardiovascular exercise per week and sessions of muscle-strengthening exercise per week (14). These have been implemented in the context of cardiac rehab for patients post-event and are encouraged for patients in the acute care phase for many other conditions such as cancer (121, 122). Furthermore, in community settings, as per WHO and ACSM guidelines, targets for physical activity and exercise are commonly promoted but underprescribed (123). The pain points which impede implementation include a lack of time and resources in the primary care setting, which a digital solution can address. We will now briefly cover the elements of DET developers should consider when building a scalable solution. Each aspect of the intervention within the treatment product should adhere to the principles of exercise science, which include: Individualisation, specificity, progression of training load, recovery, reversibility and transfer (35, 124). Importantly a clear distinction must always be made between promotion and prescription, as the former is not treatment. As described by Conroy and colleagues, “poorly designed interventions deployed via digital modes are no more than a digital placebo—they lack a defined target or active ingredient but are delivered in a shiny new capsule” (125). As such, the intervention should be developed with treatment in mind and delivered through novel, innovative approaches as an intervention.

### General physical activity

Physical activity refers to any form of repetitive movement during waking hours, typically involving large muscle groups resulting in an increase of energy expenditure. Higher levels of physical activity are associated with a reduction in morbidity and all-cause mortality, whilst an increase in sedentary time is associated with deleterious health effects (119, 126). Modern smartphones include accelerometers and gyroscopes, enabling accurate quantification of physical activity through steps taken per day. Whilst mobile applications appear effective at increasing physical activity (127), there are a number of considerations to take into account when developing a DET clinical product.

There is first the issue of pre-existing wearable technology use in patients. Wearable utilisation will differ depending on location, age and socioeconomic circumstances. In cases where a patient is already engaging with software provided with their smartwatch, monitoring their steps daily, the likelihood of achieving engagement with an additional app is questionable. Secondly, most individuals living with a chronic condition are older adults (128). It has been reported that in a cohort >65 years of age, only 7.6% use a fitness tracker, and 3.3% use a smartwatch (72). Another concern relates to the unlikelihood that individuals can remain tethered to their phone during all waking hours. Where an individual does not have a wearable and must rely on their phone to estimate steps, non-carrying time can undermine the intervention (129, 130).

A final issue relates to effect size and how the intervention meaningfully impacts health. Whilst a dose-response is present between physical activity quantified through steps and morbidity and mortality, this is cross-sectional, longitudinal evidence. There is a lack of evidence to show a causal effect on parameters such as cardiorespiratory fitness and cardiometabolic health (131). Moreover, randomized controlled trials highlight that the average increase in steps achieved through a digital intervention is only 2,000 steps per day on average (132, 133). The evidence therefore suggests that when striving for clinical impact, the DET should focus on interventions beyond steps per day, emphasising cardiovascular and resistance exercise in people living with chronic conditions.

### Cardiovascular exercise

For people living with a chronic condition, cardiovascular exercise has been shown to offer a safe and effective therapeutic benefit (35). The dose response between engagement in this mode of exercise and cardiorespiratory fitness is well established (134). In comparison to interventions emphasising daily step counts, those focusing on cardiovascular exercise offer clear advantages. First, where an individual relies on their phone as a therapeutic tool in the absence of a wearable, in order to accurately examine daily step counts during waking hours, they must have substantial contact time with the device. For example, over a 12-week period, the user must be tethered to their phone for up to 1,344 h. This is unlikely to be achieved and may set the intervention up for failure and disengagement. In contrast, striving towards 150 min of cardiovascular exercise, the contact time required with the device is far less. For example, having the patient carry their phone during each exercise session over 12 weeks equates to 30 h, which represents 98% less demand compared to the hypothetical step count centric intervention.

Not only is this intervention more feasible and likely more enjoyable for the patient without a wearable, it offers substantially more clinical impact. As has been demonstrated in traditional care, cardiovascular exercise improves several aspects of health, including cardiorespiratory fitness, cardiometabolic makers of health, psychological wellbeing and quality of life (18). As discussed, there is a need to build the intervention with risk management and clinical effectiveness in mind. After engaging in pre-participation screening and baseline assessment, the implementation of the intervention with active engagement by the patient is what drives clinical impact. DET product developers, when developing features to drive clinical impact, should emphasise commencement with the minimal effective dose to drive health gain. Whilst high intensity interval training and vigorus cardiovascular exercise offer therapeutic benefits for patients, in an undersupervised context, risk must be considered (135). For this reason, it is logical to position moderate to somewhat hard intensity cardiovascular exercise that is mode agonistic to patients and adheres to the fundamental principles of exercise science.

A logical feature to minimise the need for human intervention with the DET is a cardiovascular exercise weekly target setter. Practically, this allows for a suitable training load to be initially prescribed and then built upon over time. Here the user could self-report their accumulated minutes or sync them via their phone or wearable. Aspects of the feature necessary to drive adherence and success include automatic feedback and tracking of minutes, a visually appealing graphical display of minutes history, a goal-setting functionality and goal-achievement feedback (111). For a DET to be scalable and successful, it must not rely heavily on human intervention such as coaching. Leveraging the findings regarding adherence predictors, the following aspects should be deeply embedded into each stage of the treatment process and, in particular, where the bulk of the intervention occurs: gamification, applicability to diverse users, include those with mobility challenges, behaviour change techniques, skill building, educational content, automatic feedback and artificial intelligence.

### Muscle-strengthening exercise

Ageing is associated with a reduction in skeletal muscle mass with a concomitant decline in functional ability (91). The trajectory of this decline is mediated in part by diet and exercise (136). Muscle-strengthening exercise has been shown to favourably impact muscle, strength, power, neuromotor control and functional ability throughout the lifespan. A complication of most chronic conditions is diminished muscle health (18). It is, therefore, unsurprising that muscle-strengthening exercise has been positioned as a therapeutic tool for this population. However, for both the general public and people living with a chronic disease, achieving the guidelines of 2 muscle-stregthening exercise sessions per week is uncommon (137).

Much like cardiovascular exercise, a dose-response relationship exists between volume, load and intensity with regard to muscle-strengthening exercise (138). For DET product developers, there is a need to balance risk with clinical impact by cautiously adhering to the principles of exercise science whilst providing the patient with the necessary instructions and opportunities to develop the skill needed to perform muscle-strengthening exercises. Indeed, for most living with a chronic condition, factors such as age, health status, injury history, movement literacy, self-efficacy, along with perceptions of exercise will influence the therapeutic approach. A logical starting point is to position safe, easy-to-implement movements requiring minimal equipment that can be performed at home.

After the patient has successfully undertaken pre-participation screening and assessment and is deemed suitable to proceed, it is necessary to commence a safe, graded exercise program. In the absence of coaching supervision, there is a need to ensure that the initial training load is safe and additionally capable of changing physiology to bring about the desired adaptation (139). The inclusion of warm-up, cool down, clear demonstration and clear guidance regarding perceived exertion is necessary to ensure appropriate risk management (35). Graded progression can be achieved through manipulation of sets, reps, duration of exercise, exercise selection, range of motion, rating of perceived exertion and load (35). The experience of the patient during the intervention will determine engagement and retention. Therefore gamification, behaviour change techniques such as goal setting, automatic feedback, artificial intelligence, skill building, and educational content should all feature in the DET. Approaches involving live kinematic feedback offer novel solutions to drive engagement, enhance safety and optimise clinical outcomes. Most mHealth apps involving exercise utilise pictures as opposed to video demonstrations without necessary engagement and support features or safety advice (140). Studies show patients are willing to engage with remote digital home exercise programs that take into consideration their individual needs (141). For DET developers, for the intervention to be successful, there is a need to adhere to fundamental princples in an innovative manner when building the treatment through a human-centred approach.

## Integration into clinical workflows

Exercise therapy is recommended as a component of treatment and long-term self-care for most chronic conditions. However, the challenge is that many clinicians do not have the time or training to adequately assess and prescribe exercise therapy. The clinical effectiveness and safety of a DET solution that can assess a patient and prescribe exercise therapy is realized through adequate product development and subsequent hypothesis testing via randomized control trials. A key consideration of developers is the positioning of the solution within clinical workflows, which requires thoughtfulness in order to optimize the real-world practice of the prescribing clinician. The Chronic Care Model (CCM) is an evidence-based framework for organizing and providing care for people with chronic conditions with an emphasis on patient-centered care (15). The CCM consists of six key components of healthcare delivery which allow for the identification of how a DET can fit into clinical workflows.

1. Organization of Healthcare Delivery System: Here there is an emphasis on developing a culture, organization and mechanisms that promote safe, high-quality care in either a hospital or primary care setting. Within the CCM, the system benefits from evidence-based solutions that enhance patient outcomes. DET developers should consider the implementation of the solution early in the product development stages. Solutions that embed into the system from the perspective of integrated adjunctive care will have a far more favorable probability of adoption by clinicians. For example, in an acute care setting, a DTx offering exercise therapy to patients unable to attend phase III cardiac rehabilitation addresses an unmet need within the delivery system, improves clinical outcomes, and can enhance the decision-making of clinicians.
2. Clinical Information System: The organization of patient data can lead to efficient and effective care. A health system can utilize the technology of a DET as a DTx by monitoring patient status which can be accessed by clinicians via a portal. For example, a patient living with Type 2 Diabetes being managed at the primary care level can have their exercise, glucose monitoring and medication adherence data monitored in real-time by the prescribing clinician. This can optimize the coordination of care and delivery of care leading to better clinical outcomes and reducing patient risk.
3. Delivery System Design: This emphasizes that care delivery is effective and efficient by ensuring regular, proactive visits focusing on patient goals and including necessary follow-ups. Data obtained through self-monitoring within a DET solution can help optimize these clinical interactions. For example, a breast cancer survivor with a DET solution can monitor key aspects of health including subjectively reported lymphedema, energy, fatigue, and mood along with quantitively reported cardiovascular fitness and exercise output (minutes per week or steps per day etc.). This information can be used to enhance clinical decision-making.
4. Self-Management Support: Within the CCM there is a specific emphasis on shifting the focus from reactive to proactive care by empowering the patient. Chronic conditions must be managed for life and this requires necessary behavior change in relation to specific aspects of care. A DET can facilitate this in part through disease-specific education and upskilling the patient in relation to medication adherence along with exercise therapy. For example, a patient with osteoporosis will benefit from specific education regarding medication, basic dietary changes, and essentially long-term engagement with exercise to favorably change bone mineral density. In doing so reduces the burden on the healthcare system and ensures that clinical workflows are optimized.
5. Decision Support: Here there is an emphasis on promoting clinical care that is consistent with scientific evidence and patient preferences. There is a need to share information with patients to encourage their participation and in particular use proven provider education methods. Therefore, a DET that has been shown to be clinically effective and adheres to evidence-based practice is likely to be perceived by clinicians as a useful tool to enhance their clinician workflows, provided the DET is sensitive to all components of the CCM.
6. Community Linkage: This final component considers community resources that allow patients to identify opportunities in their locale that help them adopt healthy lifestyle changes. The power of community from the perspective of health cannot be underestimated. Whilst a DET solution alone is a potentially powerful tool for behavior change, there should be an emphasis on encouraging patients to experience community offerings for long-term behavior change that are evidence-based and cost-effective.

The CCM, therefore, aims to help inform, activate, and empower patients alongside prepared and proactive healthcare teams. Developers of DET solutions should consider the six components of the CCM in order to adequately design the solution to enhance existing clinical workflows. In doing so the clinical effectiveness of care along with risk management of the patient will be optimized.

## Conclusions

The aim of this paper was to highlight key considerations for DET product developers aiming to build both over-the-counter and prescription DTx solutions for people living with chronic conditions. There is a pressing need to optimize for patient safety and clinical impact. This can be achieved by taking a human-centered design approach in each aspect of care: pre-participation screening, assessment, exercise prescription and adherence optimization. The objective should be to first be as good as traditional care and aim to extend beyond the current limitations and pain points with novel digital solutions. There are myriad opportunities in the prevention and treatment space for DET solutions that are attractive to payers, providers, pharma companies and most importantly, patients and healthcare providers. Thoughtful approaches are required to include key stakeholders in the development process, to breakdown existing barriers, and ensure the DET solution is successfully implemented within existing ecosystems and workflows.
